# Recent Advances in Fibrosis and Scar Segmentation From Cardiac MRI: A State-of-the-Art Review and Future Perspectives

**DOI:** 10.3389/fphys.2021.709230

**Published:** 2021-08-03

**Authors:** Yinzhe Wu, Zeyu Tang, Binghuan Li, David Firmin, Guang Yang

**Affiliations:** ^1^National Heart and Lung Institute, Faculty of Medicine, Imperial College London, London, United Kingdom; ^2^Department of Bioengineering, Faculty of Engineering, Imperial College London, London, United Kingdom; ^3^Cardiovascular Biomedical Research Unit, Royal Brompton Hospital, London, United Kingdom

**Keywords:** cardiac magnetic resonance, late gadolinium enhancement, scar segmentation, deep learning, atrial fibrillation, myocardial infarction

## Abstract

Segmentation of cardiac fibrosis and scars is essential for clinical diagnosis and can provide invaluable guidance for the treatment of cardiac diseases. Late Gadolinium enhancement (LGE) cardiovascular magnetic resonance (CMR) has been successful in guiding the clinical diagnosis and treatment reliably. For LGE CMR, many methods have demonstrated success in accurately segmenting scarring regions. Co-registration with other non-contrast-agent (non-CA) modalities [e.g., balanced steady-state free precession (bSSFP) cine magnetic resonance imaging (MRI)] can further enhance the efficacy of automated segmentation of cardiac anatomies. Many conventional methods have been proposed to provide automated or semi-automated segmentation of scars. With the development of deep learning in recent years, we can also see more advanced methods that are more efficient in providing more accurate segmentations. This paper conducts a state-of-the-art review of conventional and current state-of-the-art approaches utilizing different modalities for accurate cardiac fibrosis and scar segmentation.

## 1. Introduction

Necrosis regions found in the heart (including left atrium (LA) pre-ablation fibrosis, LA post-ablation scar and left ventricle (LV) infarction), depending on the location and size, can have various implications on the cardiac conditions of the patients. For example, ventricular scars can be signs of earlier episodes of myocardial infarction (MI) (Choi et al., 2001; Krittayaphong et al., 2008; Wu et al., 2008; Larose et al., 2010). Locating and quantifying the fibrosis and scars have also been demonstrated as a valuable tool for the treatment stratification of patients with atrial fibrillation (AF) (Allessie, 2002; Boldt, 2004) or ventricular tachycardia (Ukwatta et al., 2015) and provide guidance information for the surgical or ablation based procedures (Vergara and Marrouche, 2011). Imaging of post-ablation scars may also give valuable information on treatment outcomes (Peters et al., 2007; Badger et al., 2010).

Cardiovascular magnetic resonance (CMR) has been one of the modern imaging techniques, which is widely used for qualitative and quantitative evaluation of cardiac conditions and to support diagnosis, monitoring disease progression and treatment planning (Kim et al., 2009). In particular, Late Gadolinium enhancement (LGE) CMR has been an emerging technique for locating and quantifying regions of fibrosis and scars across the LA and the LV (Peters et al., 2007; McGann et al., 2008; Oakes et al., 2009; Akkaya et al., 2013; Bisbal et al., 2014). LGE CMR has also been shown to improve ablation strategy planning, treatment stratification and prognosis by pre-ablation fibrosis quantification via clinical validations (Akoum et al., 2011). It also enabled computationally guided and personalized targeted ablation in treating AF in clinical practices (Boyle et al., 2019).

Many algorithms have been developed for the segmentation of cardiac scarring regions, and a few challenges have benchmarked some of the high-performing methodologies (Table 1). Among these, 2-SD (standard deviation) has been advocated by the official guidelines (Kramer et al., 2013), while the full width at half maximum (FWHM) technique has been advocated as the most reproducible method to segment ventricular scars (Flett et al., 2011) (see Section 3.2 for descriptions of 2-SD and FWHM methods). As these algorithms are usually based on successful segmentation of the corresponding anatomical regions beforehand as an accurate initialization, there has also been rising attention to the automated segmentation of LA and LV anatomy from the LGE CMR images (Table 1).

**TABLE 1:** List of challenges in segmentation of LV and LA anatomy and scar in LGE CMR.

Year	Challenge/Dataset	Conference (MICCAI/IBSI etc.)	Modality (data size n)	Target	Pathology
2012	LV scar segmentation challenge (Karim et al., 2016)	MICCAI	LGE MRI (30)	LV scar	MI
2013	LA scar segmentation challenge (Karim et al., 2013)	ISBI	LGE MRI (30)	LA scar	AF
2018	LA segmentation challenge (Xiong et al., 2021)	MICCAI	LGE MRI (150)	LA cavity	AF
2019	Multi-sequence Cardiac MR Segmentation Challenge (MS-CMR) (MS-CMR Challenge, 2019)	MICCAI	LGE MRI, T2 MRI, bSSFP MRI (45, coregistered)	LV blood pool, RV blood pool, LV myocardium	MI
2020	Myocardial pathology segmentation combining multi-sequence CMR (MyoPS) (MyoPS Challenge, 2020)	MICCAI	LGE MRI, T2 MRI, bSSFP MRI (45, coregistered)	LV blood pool, RV blood pool, LV normal myocardium, LV myocardial oedema, LV myocardial scar	MI

With the development of artificial intelligence techniques, we can observe a rising number of various deep learning models using convolutional neural networks [e.g., fully connected neural network (FCNN) (Szegedy et al., 2016) and U-Net (Ronneberger et al., 2015)], which have demonstrated encouraging results in segmentations of cardiac substructures in recent years (Chen C. et al., 2020). It has also been found that deep learning can be directly applied to scar segmentation as a fully automated end-to-end solution for the input LGE CMR images. With co-registration of different modalities together and deep learning based transfer learning, the combination of LGE CMR with other CMR imaging modalities [e.g., balanced steady-state free precession (bSSFP)] may further improve the efficacy and efficiency of the segmentation results.

The use of Gadolinium-based contrast agent (GBCA) has led to concerns over the patient’s safety, particularly for the patient with renal impairments (Ledneva et al., 2009). With deep learning based methods, cardiac scarring regions can now be localized and quantified in non-Gadolinium enhanced CMR images without GBCA injections (Zhang et al., 2019).

As all pre-2016 and pre-2013 cardiac scarring segmentation have been carefully benchmarked and summarized by Karim et al. (2013, 2016), this paper instead focuses on the survey of all post-2016 methodologies in fibrosis and scars delineation and segmentation of the LA and LV anatomy from LGE CMR images. This study also discusses the potential use of the modalities other than LGE CMR in locating and quantifying the scars.

### 1.1 Search Criteria

**FIGURE 1:**
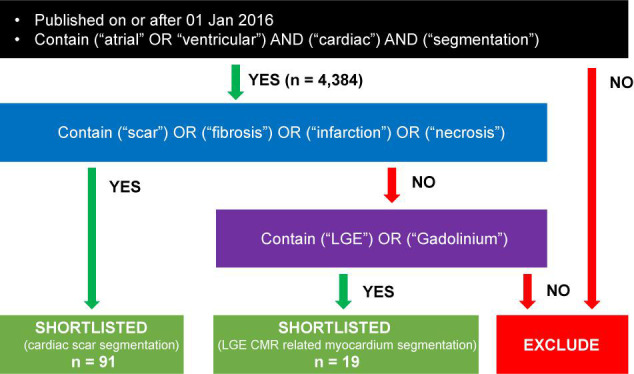
Flowchart to demonstrate the search criterion.

To identify related contributions, search engines like Scopus and Google Scholar were queried for papers on or after 01 Jan 2016 containing (“atrial” OR “ventricular”) and (“cardiac”) and (“segmentation”) with or without (“scar”) in their titles or abstracts. Papers that do not primarily focus on the segmentation of cardiac scar or scar-related cardiac anatomy were excluded. Each paper was reviewed and agreed upon by at least two of us (Y.W., Z.T., B.L.) before inclusion. We found 4,384 papers from the search engines and shortlisted 110 of them following the criterion above (Figure 1). After full-text screening for their relevances to the topic, we eventually included 47 of them into this study. The last update to the included papers was on 13 May 2021.

## 2. Imaging Modalities

### 2.1 LGE CMR

Fibrosis found in LA are signs of atrial structural remodeling and can be considered as a major risk factor in the progression of the atrial fibrillation (AF) (Allessie, 2002; Boldt, 2004), where the identification of scarring and fibrosis regions in LA has been crucial for diagnosis, prognosis and treatment planning. Native pre-ablation fibrosis can be a sign of AF recurrence (Oakes et al., 2009), and post-ablation detection of ablation induced scars can facilitate the identification of post-ablation ablation line gaps, which is the main reason of ablation failures (Peters et al., 2007; Badger et al., 2010). In contrast to the traditional method of the electro-anatomical mapping (EAM) system, which is an invasive technique in localization of the atrial scar and the fibrosis with suboptimal accuracy (Zhong et al., 2007; Schmidt et al., 2009), LGE CMR enables the atrial scarring and fibrosis regions to be localized and quantified non-invasively without ionizing radiation. LGE CMR employs the slow washout kinetics of Gadolinium in these regions to highlight these scarring and fibrosis regions (Peters et al., 2007; McGann et al., 2008; Oakes et al., 2009; Akkaya et al., 2013; Bisbal et al., 2014).

In addition to the atrium, LGE CMR has also been considered as a gold-standard modality for the assessment and quantification of the scarring regions in the left ventricle (Simonetti et al., 2001; Wu et al., 2001; Wagner et al., 2003a; Hendel et al., 2006), where fibrotic and scarring regions found can be considered as a sign of earlier or current episodes of the MI (Choi et al., 2001; Krittayaphong et al., 2008; Wu et al., 2008; Larose et al., 2010). In addition to MI, with growing prognostic evidence, LGE has been successful in the identification of scarring regions in cardiomyopathy, inflammatory and infiltrative conditions (Wagner et al., 2003b; Maceira et al., 2005; Smedema et al., 2005; Flett et al., 2009).

**FIGURE 2:**
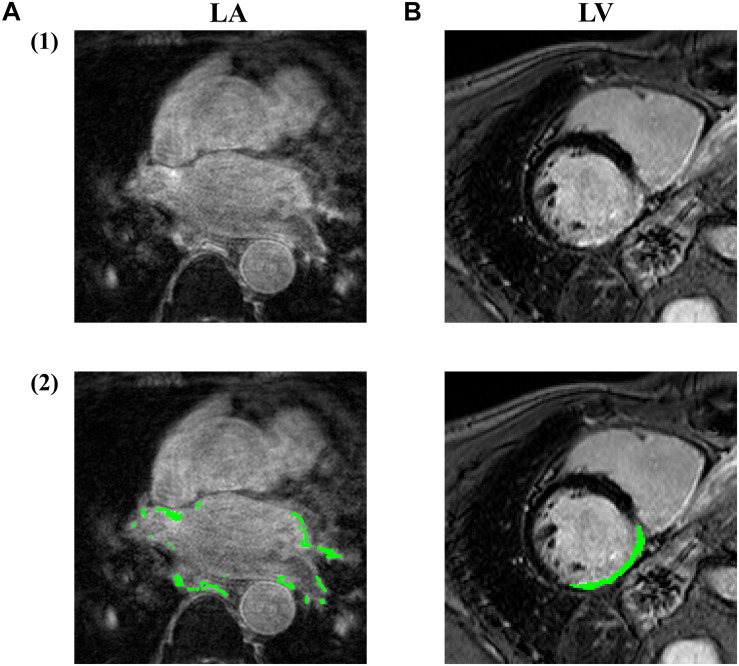
Examples of LGE CMR images acquired at **(A)** LA and **(B)** LV, with the fibrosis/infarction regions highlighted in green. By comparing **(A2)** and **(B2)**, we can see the fibrosis region in LA is rather more discrete and thinner compared to LV infarction, making LA fibrosis regions more difficult to be accurately fully localized and quantified. Image source: **(A)** was extracted from pre-ablation CMR images in ISBI 2013 cDERMIS dataset (http://www.cardiacatlas.org/challenges/left-atrium-fibrosis-and-scar-segmentation-challenge/). **(B)** was extracted from MICCAI 2012 Ventricular Infarct Segmentation challenge dataset (http://www.cardiacatlas.org/challenges/ventricular-infarct -segmentation/).

However, the LGE CMR modality often suffers from poor image qualities, which may be due to residual respiratory motions, variabilities in the heart rate and gadolinium wash-out during the currently long acquisition time (Yang et al., 2017). Particularly, the spatial resolution of the left atrium in the LGE CMR image is limited (To et al., 2011), considering the thin transmural thickness of the atrial wall [mean = 2.2–2.5 mm (Saìnchez-Quintana et al., 2005)] (Figure 2). The variable anatomical morphological shapes of the LA and pulmonary veins (PV) also impose an additional challenge to the LGE CMR segmentations. To improve the visualization of these scar regions, we can see a successful attempt by maximum intensity projection (MIP) to enhance intensities on post-ablation LA LGE CMR (Knowles et al., 2010). Moreover, some irrelevant cardiac substructures may be highlighted in LGE CMR images as well, in addition to the scarring and fibrosis regions. These may be due to, for example, the navigator beam artifact, which is often seen near the right PV, Gadolinium uptake by the aortic wall and valves, and confounded enhancement in the spine, esophagus, etc. (Karim et al., 2013; Yang et al., 2017). As a result, these can lead to a poor result in the delineation of LA and LV scar or fibrosis regions and even a significant amount of false positives in segmentations of these structures and regions.

In addition, although LGE CMR has been successful in being the gold standard reference technique for AF and MI, including LGE in MRI significantly extends the scanning time. There have been also increasingly growing concerns regarding the safety of the Gadolinium based contrast agent used, particularly for the patient with renal impairments (Ledneva et al., 2009).

### 2.2 LGE CMR With Other Modalities

**FIGURE 3:**
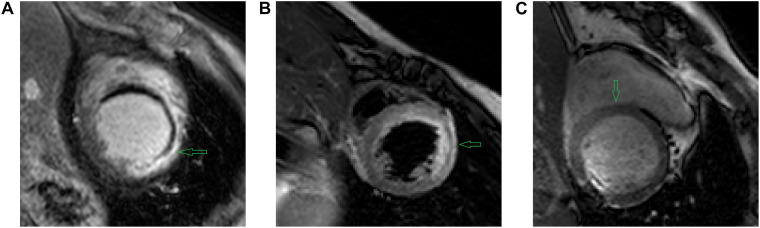
Example images using different CMR sequences acquired by **(A)** LGE CMR **(B)** T2 CMR **(C)** bSSFP CMR. As denoted by the green arrows, we can see **(A)** LGE CMR accentuates the scar tissue by high intensities on the images; **(B)** T2 CMR accentuates myocardial oedema by high intensities on the image; and **(C)** bSSFP CMR shows the distinct endo- and epi-cardial boundary of the myocardium clearly on the image. Image source: **(A–C)** extracted from the MS-CMR open challenge dataset (MS-CMR Challenge, 2019).

In addition to LGE MRI, which could highlight the scarring regions, segmentation of the anatomy and scarring regions can also utilize other modalities (Figure 3) to further improve the accuracy if applied with LGR CMR by co-registering different modalities together (Zhuang, 2019).

There have been challenges benchmarking a range of algorithms for the cross-modality fusion based segmentation of anatomy, scar and oedema.

(1) MS-CMR challenge (MS-CMR Challenge, 2019; Pop et al., 2020) presented a range of algorithms taking multiple modalities in to further improve the segmentation accuracy of LV myocardium, LV blood cavity and RV.
(2) MyoPS challenge (MyoPS Challenge, 2020; Zhuang and Li, 2020) presented algorithms to delineate LV myocardium with scarring and oedema.

Other modalities and sequences can include:

(1) Magnetic resonance angiography (MRA) sequence – to image LA and PV with high contrasts, which has been demonstrated by Tao et al. (2016) to improve the error distance in segmenting LA anatomy to within 1.5 mm. However, MRA is usually ungated and usually acquired in an inspiratory breath-hold, making anatomy delineated from MRA significantly distorted from LGE CMR.
(2) Balanced steady-state free precession (bSSFP) – provides a clear boundary between the myocardium and blood cavity under movements, which is usually respiratory and cardiac gated. It can offer cine CMR with a uniform texture.
(3) T2 – high intensities in T2 presents myocardial oedema with high specificity and sensitivity (Gannon et al., 2019), T2 could be helpful in segmenting myocardial oedema and scar simultaneously if incorporated with LGE-CMR and bSSFP (Zhu et al., 2017). Identification of oedema on CMR can help clinicians to differentiate between acute and remote myocardial infarction (Friedrich, 2017). The presence of oedema in patients without extensive irreversible injury (e.g., scar) can serve as a marker for clinicians to predict the recovery of LV systolic functions (Vermes et al., 2014).

## 3. Conventional Methods

Conventionally, a two-stage approach is adopted in the identification and evaluation of fibrotic and scarring tissue – (1) segmentation of the relevant anatomical structure (LA and PV in the case of LA fibrosis/scar segmentation and LV in the case of LV infarction segmentation) and (2) then segmentation of the fibrotic and scarring regions. This two-stage approach is particularly beneficial for LA and PV, as LA and PV are highly morphological variables and relatively small in size. We shall then elaborate on the recent developments of methodologies for each of them.

### 3.1 Segmentation of Anatomical Structures

The delineation of anatomical structures, e.g., LA and LV wall, from others can be difficult in LGE CMR images. In LGE scarring tissues are significantly enhanced while the signals from the healthy tissues are attenuated (Keegan et al., 2015), making the segmentation of LA, PV and LV anatomical structures very challenging.

#### 3.1.1 Why Is Accurate Segmentation of Anatomical Structure Necessary Before Scar Segmentation?

Accurate segmentation of the anatomy (LA or LV wall) is essential as it gives an accurate initialization for the scar segmentation. Therefore, traditionally, the segmentation of these structures were all done manually.

We could see in the cDEMRIS challenge in ISBI 2012 (Karim et al., 2013) algorithms with manually initialized LA segmentation showed significantly better performance than Others. It demonstrated the need for an accurate anatomy segmentation ahead of the scar segmentation along with Rajchl et al. (2015). Moccia et al. (2018) also demonstrated that manual and accurate segmentation of the LV wall could improve the deep learning based segmentation of the LV infarction.

#### 3.1.2 Conventional Methods in Segmenting Anatomical Structures

In the early 21st century, radiologists looked between LGE CMR and cine CMR back and forth to delineate the myocardium region. To mimic that, we can see methods in the first decade and early second decade of this century utilizing both LGE and cine modalities by, for example, non-rigid registration to achieve high accuracy in segmentation of myocardium over LGE CMR (Dikici et al., 2004; Ciofolo et al., 2008; Wei et al., 2011, 2013). However, by doing so, the result may suffer from registration misalignment between LGE and cine modalities and the model may be computationally demanding. As such, from 2014 we can see methods that are less computationally demanding and using LGE modality only (Albà et al., 2014; Kurzendorfer et al., 2017a,b,c).

Conventional methods in medical image segmentation usually have limited efficacy. Representative methods are summarized in Table 2, which mainly include the following methodologies.

(1) Random forest (Kurzendorfer et al., 2017b).
(2) Image registration (Kurzendorfer et al., 2017c).
(3) Markov random field (MRF) model (Albà et al., 2014).
(4) Atlas-based modeling with active contour model (Kurzendorfer et al., 2017a).
(5) Principal component analysis (PCA) technique (Kurzendorfer et al., 2017c).

**TABLE 2:** Summary of representative conventional methodologies for segmentation of the myocardium on LGE-MRI.

Reference	Modalities	Methodology description	Pros	Cons	Quantitative result (myocardium)	Dataset
Dikici et al., 2004	LGE MRI, cine MRI	(1) Define LV border – non-rigid registration of cine and LGE MRI (2) LV pixel classification – SVM	Automatic segmentation of LGE-MRI with CINE-MRI information	No longitudinal axis (LAX) consideration, resulting in inter-slice misalignment; Need to register with other modality (CINE MRI)	Average contour pixel location error = 1.54 pixel	Private (LV LGE + cine MRI, *n* = 45)
Ciofolo et al., 2008	LGE MRI, cine MRI	2D segmentation with a geometrical template (LGE only) and 3D mesh alignment (LGE + CINE)	Overcome non-homogeneous intensity of the myocardium in LGE infarcted regions	Meshes focus only on features in the SAX slices, no inter-slice consideration and thus inter-slice misalignment; Need to register with other modality (CINE MRI)	ASD = 2.2 mm (endocardial), 2.0 mm (epicardial)	Private (LV LGE + cine MRI, *n* = 27)
Wei et al., 2011	LGE MRI, cine MRI	(1) Affine transformation estimation (2) non-rigid registration of LGE and cine MRI (3) myocardial contour generation by simplex mesh geometry	Utilize information better in connecting cine and LGE MRI	No LAX consideration, resulting in inter-slice misalignment; Need to register with other modality (CINE MRI)	Mean Dice = 0.8249; ASD = 0.97 pixel (endocardial), 0.93 pixel (epicardial)	Private (LV LGE + cine MRI, *n* = 10)
Wei et al., 2013	LGE MRI, cine MRI	Translational registration of LGE and cine MRI data; 3D non-rigid deformation of the myocardial meshes by both short axis (SAX) and longitudinal axis (LAX) data	Consistent and robust segmentation; Consider both SAX and LAX data to reduce interslice misalignment	Need to register with other modality (CINE MRI)	Mean Dice = 0.9409; ASD = 0.67 mm (endocardial), 0.69 mm (epicardial)	Private (LV LGE + cine MRI, *n* = 21)
Albà et al., 2014	LGE MRI	Slice-by-slice graph cuts (GC) with interslice and shape constraints	Impose morphological constraints that are common across MRI sequences – no need for subject-specific tuning or for user initialization and generalizable for other sequences (CINE-MRI); Achieve robustness to variations in grey-level appearance and to image inhomogeneities – more robust to the presence of abnormalities; Consider interslice interactions; No need to register with other modality (e.g., bSSFP cine MRI)	Give poorer result when generalized to CINE-MRI (due to many artefacts in the dataset tested)	Mean Dice = 0.81; ASD = 1.83 mm (endocardial), 2.38 mm (epicardial)	Private (LV LGE MRI, *n* = 20)
Kurzendorfer et al., 2017c	LGE MRI	(1) LV localization – image registration (2) short axis estimation – principal component analysis (PCA) (3) endocardial refinement – a minimal cost path search (MCP) in polar space using the edge and scar information (4) epicardial refinement - by shape and inter-slice smoothness constraints (5) surface extraction – 3D mesh generation by marching cube algorithm (Lorensen and Cline, 1987)	Fast speed and low computational workload by using simple texture features; Consider image data along the longitudinal axis in addition to the short axis, improving inter-slice smoothness and avoid inter-slice shift; No need to register with other modality (e.g., bSSFP cine MRI)	Poor performance in apex and LV outflow tract, poor accuracy in basal regions; Since this method is texture based, the distribution of scar and the small size of the atrium adversely affect its performance	Mean Dice = 0.92; ASD = 1.35 mm	Private (LV LGE MRI, *n* = 30)
Kurzendorfer et al., 2017a	LGE MRI	(1) LV detection – circular Hough transforms (2) LV blood pool detection – morphological active contours approach without edges (MACWE) (3) endocardial boundary extraction – a minimal cost path search (MCP) in polar space using the edge and scar information (4) epicardial boundary extraction – by edge information while considering endocardial contour extracted	Fast speed and low computational workload by using simple texture features; No need to register with other modality (e.g., bSSFP cine MRI)	Poor performance in apex and LV outflow tract, poor accuracy in basal regions; Since this method is texture based, distribution of scar adversely affect its performance	Mean Dice = 0.85 (endocardial), 0.84 (epicardial); ASD = 2.54 mm (endocardial), 3.32 mm (epicardial)	Private (LV LGE MRI, *n* = 26)
Kurzendorfer et al., 2017b	LGE MRI	(1) LV detection – circular Hough transforms, Otsu thresholding and circularity measures (2) ROI detection – morphological active contours approach without edges (MACWE) (3) endocardial boundary extraction – random forest classifier (4) epicardial boundary extraction – minimal cost path search to the boundary cost array in polar space	Fast speed and low computational workload by using simple texture features; No need to register with other modality (e.g., bSSFP cine MRI)	Poor performance in apex and LV outflow tract, resulting in poor accuracy in basal regions and poor ASD result	Mean Dice = 0.83 (endocardial), 0.83 (epicardial); ASD = 3.55 mm (endocardial), 4.12 mm (epicardial)	Private (LV LGE MRI, *n* = 100)

*ASD: average surface distance.*

For LA, in particular, the methods involving pre-defined shape priors (Zhu et al., 2013; Veni et al., 2017) often suffer from relatively poor error distance, which is more than 1–2 mm required (Xiong et al., 2021) under the clinical setting considering the thin LA wall (Zhao et al., 2017). However, one of them reported a relatively high Dice score (79%) (Zhu et al., 2013).

### 3.2 Segmentation of Scarring Regions

Upon successful segmentation of the anatomy, the scarring regions can be identified by a range of approaches. These approaches can be mainly divided into the following categories: threshold based methods, classification methods, or the combination of both.

#### 3.2.1 Fixed Threshold Based Methods (n-SD and FWHM)

Traditionally, the scarring regions can be detected as they are accentuated in LGE CMR. Among a range of conventional techniques, 2-SD has been advocated by official guidelines (Kramer et al., 2013), while the full width at half maximum (FWHM) technique has been advocated as the most reproducible method to segment ventricular scars (Flett et al., 2011).

2-SD and FWHM are both fixed threshold methods in segmenting the scarring region, where pixels with intensities above a fixed threshold would be labeled as the scar. 2-SD or even n-SD methods define such threshold as the sum of the mean and two or n standard deviations of signal intensities in a remote reference region, whereas FWHM defines such threshold as the half of the maximum signal intensity within the scar.

Karim et al. (2016) evaluated 2, 3, 4, 5, 6 -SD and FWHM methods on a public human LV infarct dataset and showed that FWHM superseded all n-SD methods tested by its Dice Scores and that the Dice Scores went slightly higher with the threshold rising from 2 to 6 -SD. However, it is not the case when Karim et al. (2013) evaluated 2, 3, 4 -SD and FWHM on a public human LA fibrosis/scar dataset. For pre-ablation LA fibrosis, FWHM performed much worse than all n-SD methods tested. For post-ablation LA scar, FWHM gave similar Dice Scores as 2-SD’s with 3, 4, 6 -SD methods’ Dice Scores much lower than these two.

However, these fixed-threshold techniques, including n-SD and FWHM, are unlikely to handle variations well (Oakes et al., 2009). The variations can come from two sources – scar itself and external circumstances. Scars are highly variable in their morphology and their brightness distribution on LGE CMR. Varied external factors including resolution, contrast, signal-to-noise ratio (SNR), inversion time and surface coil intensity variation can also adversely impact the accuracy of the segmentation. This is particularly the case for pulmonary veins, which are highly morphological variables.

#### 3.2.2 Conventional Adaptive Methods

An LV scar segmentation challenge (Karim et al., 2016) organized in MICCAI 2012 and LA scar segmentation (Karim et al., 2013) challenge organized in ISBI 2013 carefully benchmarked and summarized the majority of the pre-2013 conventional methods. In the LV segmentation challenge in 2012, it showed all of the algorithms benchmarked did not exhibit superiority against FWHM, although they did perform better than n-SD methods.

##### 3.2.2.1 Adaptive thresholding based methods

Conventional threshold based approaches are summarized in Table 3A, which mainly include the following methodologies.

(1) Otsu thresholding (Otsu, 1979; Tao et al., 2010).
(2) Histogram analysis (Karim et al., 2013).
(3) Hysteresis thresholding (Karim et al., 2013).
(4) Constrained watershed segmentation (Hennemuth et al., 2008).

**TABLE 3:** Summary of representative conventional methodologies for segmentation of cardiac scar and fibrosis regions on LGE-MRI.

Type of method	Reference	Method Description	Pros	Cons	Quantitative result (scar/fibrosis)	Dataset
(A) Thresholding	Hennemuth et al., 2008	Histogram analysis with constrained watershed segmentation	Automatic threshold determination; No training (supervision) needed;	Based on fixed models – mismatches occur for some cases	*	Private (LGE MRI, *n* = 21)
	Tao et al., 2010	Otsu thresholding (Otsu, 1979) Refine segmentation – (accept false rejection) connectivity filtering and (reject false acceptance) region growing	Automatic threshold determination; No training (supervision) needed; No specific density model assumed – no overfitting; Region growing technique can be useful for small MI	Connectivity filtering and region growing may not be suitable for discrete LA fibrosis regions	Mean Dice = 0.83	Private (LV LGE MRI, *n* = 20)
	Cates et al. (2013) (part of Karim et al., 2013)	Histogram analysis and simple thresholding	Simple and accurate processing	Time consuming (require manual work); Manual variance may be significant for the thin LA wall	Median Dice = 0.42 (pre-ablation); Median Dice = 0.78 (post-ablation)	ISBI cDERMIS 2013 (Karim et al., 2013) [LA LGE MRI, *n* = 30 (pre-ablation), 30 (post-ablation)]
	Bai et al. (2013) (part of Karim et al., 2013)	Hysteresis thresholding (Canny, 1986)	Coherent segmentation (adjacent faint scar sections can still be segmented)	Fixed parameterized model relying on empirical data	Median Dice = 0.37 (pre-ablation); Median Dice = 0.76 (post-ablation)	ISBI cDERMIS 2013 (Karim et al., 2013) [LA LGE MRI, *n* = 30 (pre-ablation), 30 (post-ablation)]
(B) Classification	Perry et al. (2013) (part of Karim et al., 2013)	K-means clustering	Relatively higher performance in pre-ablation fibrosis segmentation result benchmarking; No training (supervision) needed	Cluster number to be determined beforehand; Variance in LA scar segmented	Median Dice = 0.45 (pre-ablation); Median Dice = 0.72 (post-ablation)	ISBI cDERMIS 2013 (Karim et al., 2013) [LA LGE MRI, *n* = 30 (pre-ablation), 30 (post-ablation)]
	Karim et al. (2013) (part of Karim et al., 2013)	Markov random fields (MRF) model with graph-cuts	Relatively higher performance in pre-ablation fibrosis result benchmarking;	Require necessary post-processing steps to refine clustering	Median Dice = 0.30 (pre-ablation); Median Dice = 0.78 (post-ablation)	ISBI cDERMIS 2013 (Karim et al., 2013) [LA LGE MRI, *n* = 30 (pre-ablation), 30 (post-ablation)]
	Gao et al. (2013) (part of Karim et al., 2013)	Active contour with expectation-maximization (EM)-fitting	Counteract region leaking problem in region growing techniques	Fixed number of Gaussian mixtures in model	Median Dice = 0.42 (pre-ablation); Median Dice = 0.78 (post-ablation)	ISBI cDERMIS 2013 (Karim et al., 2013) [LA LGE MRI, *n* = 15 (post-ablation)]
	Karim et al., 2014	Graph cuts	Does not requires manual outlining of base-line healthy myocardium	Require additional modality (bSSFP)	*	Private (LA LGE + bSSFP MRI, *n* = 15)
	Yang et al., 2018b	Simple linear iterative Clustering (SLIC) + support vector machine	Fully automatic scar segmentation; Able to complement minor flaws in manual annotation	Require collection of b-SSFP modality; Supervised learning – need paired manual labels for training	Mean Dice = 0.79	Private [LA LGE + bSSFP MRI, *n* = 11 (pre-ablation), 26 (post-ablation)]
	Kurzendorfer et al., 2018	Fractal Analysis and Random Forest Classification	Utilize texture information in addition to clustering	Require accurate segmentation of the myocardium	Mean Dice = 0.66	Private (LV LGE MRI, *n* = 30)

*Overall quantitative metric for the whole result population was not found. Please refer to the original article for more information of the result.

##### 3.2.2.2 Classification based methods

In addition, conventional classification approaches are summarized in Table 3B, which mainly include the following methodologies.

(1) K-means clustering (Karim et al., 2013).
(2) Graph cuts (Karim et al., 2013, 2014).
(3) Active contour with EM-fitting (Karim et al., 2013).
(4) Simple linear iterative clustering (SLIC) and support vector machine (Yang et al., 2018b).
(5) Random forest classification (Kurzendorfer et al., 2018).

## 4. Deep Learning Based Methods

Deep learning based methods are constructed from deep artificial neural networks. In this section, we will briefly introduce the common types of artificial neural networks (ANNs) and then focus on their variants targeting cardiac anatomy and scar segmentations. The authors would also like to recommend interested readers to refer to Goodfellow et al. (2016) for more detailed explanations and mathematical illustrations of these networks and Chen C. et al. (2020) for more thorough demonstrations of these networks in general cardiac imaging analysis.

### 4.1 Neural Networks of Deep Learning in Image Analysis

Convoluted neural networks (CNNs), particularly fully convoluted neural networks (FCNNs), have demonstrated success in delineating anatomical structures in medical images (Shelhamer et al., 2017), especially in cardiac MR (Chen C. et al., 2020). Successful examples include ResNet (Szegedy et al., 2016), U-Net (Ronneberger et al., 2015), and etc. U-Net (Ronneberger et al., 2015), in particular, has been known for its ability to gather latent information in medical image analysis and thus to gain better performance in segmentation, which has become the most popular CNN backbone architecture, especially after demonstrating success in the ISBI cell tracking challenge in 2015.

The recurrent neural network (RNN) is another type of ANNs. The RNN is rather more useful in processing sequential data, as it could ‘memorize’ past data and utilize its ‘memory’ to assist with its current prediction. Widely used structures of RNNs include long-short-term memory (LSTM) (Hochreiter and Schmidhuber, 1997) and gated recurrent unit (GRU) (Cho et al., 2014).

Autoencoders (AEs) are also a type of ANNs, which are able to learn latent features of imaging data. Unlike CNNs and RNNs, AEs learn these features without supervision. With latent features gathered by AEs, it could be used to guide the segmentation of medical images (Oktay et al., 2016; Yue et al., 2019).

Generative Adversarial Networks (GANs) was initially proposed for image synthesis (Goodfellow et al., 2014). With its two-player model structure (a generator network to give a synthesized image and a discriminator network to try to differentiate that synthesized image from a true image), the model can enhance the resolution of the synthesized image by adversarial training. The GAN could also be used for segmentation, where its discriminator network would rather attempt to see if the output label is in an anatomically plausible shape (Luc et al., 2016).

### 4.2 Segmentation of Anatomical Structures

#### 4.2.1 Why Use Deep Learning in the Anatomical Structure Segmentation?

There are a few challenges recently organized to benchmark the new methodologies proposed for the cardiac anatomy segmentation – 2018 LA Segmentation Challenge in MICCAI 2018 (LASC’18) (Xiong et al., 2021) for LA, MS-CMR (MS-CMR Challenge, 2019; Pop et al., 2020) in MICCAI 2019, and MyoPS 2020 (MyoPS Challenge, 2020; Zhuang and Li, 2020) in MICCAI 2020 for LV. With the recent development in deep learning, we can observe a range of methodologies developed for LA and LV segmentation in LGE CMR (Jamart et al., 2020).

In particular, in LASC’18, all deep learning methods had their mean surface distance in LA wall segmentation below 1.7 mm, with the minimum mean value of 0.748 mm. This demonstrated the efficacy of the deep learning based methods by the surface distance, which is required to be less than 1–2 mm under the clinical setting (Xiong et al., 2021).

#### 4.2.2 Deep Learning Methodologies in the Anatomical Structure Segmentation

Successful networks demonstrating success in delineating anatomical structures include VGG-net (Simonyan and Zisserman, 2014), U-Net (Zabihollahy et al., 2019b), and V-Net (Milletari et al., 2016). To further exploit the information on the z-axis, LSTM and its variants (Yang et al., 2018a; Zhang et al., 2020) and dilated residual learning blocks (Yang et al., 2018a) can be introduced to the widely used U-Net.

On top of the U-Net, Xiong et al. (2019) proposed a dual path U-Net variant, which is demonstrated to have the best Dice Score (0.942) followed by VGGNet (0.864) in their benchmarking of a range of popular CNNs including the original U-Net and one non-deep-learning based method (Zhu et al., 2013) in LA segmentation. Multi-view learning, incorporating axial, sagittal and coronal views together, gave superior performance compared to models based on one view only (Xiao et al., 2020).

On the contrary, further research showed that structural variations in U-Net are unlikely to cause a significant improvement of its performance in LA segmentation from LGE CMR (Wang et al., 2019), and that deep supervision and attention blocks are unlikely to further improve LA segmentation performance either (Borra et al., 2020b).

In addition to these supervised learning based methods, Chen J. et al. (2019) proposed a feature-matching based semi-supervised learning technique to further improve the segmentation efficacy.

All the methods discussed above are summarized in Table 4.

**TABLE 4:** Summary of representative deep learning based methodologies for segmentation of the myocardium on LGE-MRI.

Reference	Model backbone	Method description	Pros/cons	Quantitative result (myocardium)			Dataset
Zabihollahy et al., 2019b	U-Net	Standard U-Net	Fast processing; deep latent network	Mean Dice = 0.8661			Private (LV LGE MRI, *n* = 24)
Zhang et al., 2020	U-Net	U-Net with bidirectional convolutional LSTM	Process spatial sequential information	Mean Dice = 0.906			LASC’18 (Xiong et al., 2021) (LA LGE MRI, *n* = 100)
Yang et al., 2018a	U-Net	U-Net with multiview sequential learning via convolutional LSTM and dilated residual learning	Process spatial sequential information on all 3 spatial axes	Mean Dice = 0.897			Private (LA LGE MRI, *n* = 100)
Xiong et al., 2019	FCNN	Dual-path FCNN concerning both local and global view	Mitigate class imbalance; Less input image size – save GPU memory	Dice = 0.942	Benchmarking (Dice)		Private [LA LGE MRI, *n* = 40 (pre-ablation), 70 (post-ablation)]
					U-Net (Ronneberger et al., 2015)	0.642	
					Dilated U-Net (Men et al., 2017)	0.687	
					VGGNet (Men et al., 2017)	0.684	
					Inception (Szegedy et al., 2015)	0.792	
					ResNet (He et al., 2016)	0.804	
					DCN-8 (Long et al., 2015)	0.558	
					DeconvNet (Noh et al., 2015)	0.500	
					SegNet (Badrinarayanan et al., 2017)	0.656	
					V-Net (Milletari et al., 2016)	0.696	
					DeepOrgan (Roth et al., 2015)	0.632	
					Zhu et al., 2013	0.821	
Xiao et al., 2020	FCNN	3D FCNN with 3D view fusion	Process spatial information on all 3 spatial axes volumetrically; Greater amount of GPU memory occupied	Dice = 0.912			LASC’18 (Xiong et al., 2021) (LA LGE MRI, *n* = 100)
Chen J. et al., 2019	Double-sided FCNN	Semi-supervised learning – discriminative feature learning via double-sided domain adaptation	Achieve a fusion of the feature spaces of labeled data and unlabeled data to achieve semi-supervision	Mean Dice = 0.9078			Private (LA LGE MRI, two-center, n1 = 175, n2 = 94)

*We included the benchmarking quantitative results from Xiong et al. (2019) for readers’ interests, as they covered nearly all popular deep learning models for general image processing.*

### 4.3 Segmentation of Scarring Regions

We can observe a range of deep learning based methods in segmenting scars (Table 5).

**TABLE 5:** Summary of representative deep learning based methodologies for segmentation of cardiac scar and fibrosis regions on LGE-MRI.

LA/LV	Reference	Model backbone	Model description	Pros/Cons	Quantitative results (scar/fibrosis)	Dataset
(A) LA	Yang et al., 2017	Auto Encoder	Stacked Sparse Auto-Encoders	Significantly higher accuracy; Misenhancement in valves, etc. can cause false positive; Hyper-parameter sensitive	Mean Dice = 0.82	Private [LA LGE MRI, *n* = 10 (pre-ablation), 10 (post-ablation)]
	Li et al., 2020	CNN	Graph-cuts framework based on multi-scale CNN	Multi-scale consideration enables both local and global feature extraction; Surface projection mitigate difficulty in accurate LA wall delineation; Require collection of b-SSFP	Mean Dice = 0.898	Private [LA + bSSFP, LGE MRI, *n* = 58 (post-ablation)]
(B) LV	Moccia et al., 2018	E-Net	E-Net on manually segmented myocardium region only	Significantly higher accuracy; Require manual intervention in myocardium segmentation	Dice = 0.86	Private (LV LGE MRI, *n* = 30)
	Moccia et al., 2019	FCNN	FCNN on manually segmented myocardium region only	Significantly higher accuracy; Require manual intervention in myocardium segmentation	Median Dice = 0.7125	Private (LV LGE MRI, *n* = 30)
	Zabihollahy et al., 2020	U-Net	Cascaded multi-view U-Net via majority vote multi-view fusion	Consider sequential spatial information on all three axes	Median Dice = 0.8861	Private (LV LGE MRI, *n* = 34)

#### 4.3.1 LA Scar Segmentation Models

For LA (Table 5A), Yang et al. (2017) proposed a deep learning based method using Stacked Sparse Auto-Encoders to delineate the LA fibrosis region, which is based on accurate anatomical structure delineation. Li et al. (2020) proposed a graph-cuts framework based on multi-scale CNN to further incorporate local and global texture information of the images.

#### 4.3.2 LV Scar Segmentation Models

For LV (Table 5B), E-Net (Moccia et al., 2018) and FCNN (Moccia et al., 2019) were demonstrated for its high accuracy if with manually segmented LV walls. Then, multi-view U-Net has also been developed in segmenting the scar in a cascaded way (Zabihollahy et al., 2020).

### 4.4 End-to-End Automated Fibrosis and Scar Segmentation

#### 4.4.1 Development of End-To-End Scar Segmentation Models Instead of Staged Segmentation Networks

With more recent developments of deep learning, the models can extract further latent information from the LGE CMR images and segment the scar directly from LGE CMR images without acquiring accurate segmentation of the relevant cardiac anatomical structures (e.g., LA wall) in advance while maintaining the accuracy. There has also been a range of methods (Table 6) that can complete the segmentation of both the anatomy of cardiac chambers and the scar simultaneously (referred to as “two tasks” below). This is particularly the case for LV, where there is much less variability in its anatomical shape.

**TABLE 6:** Summary of representative end-to-end deep learning based methodologies for segmentation of cardiac scar and fibrosis regions on LGE-MRI.

LA/LV	Reference	Model backbone	Model description	Pros/Cons	Quantitative results (scar/fibrosis)	Dataset
(A) LA	Yang et al., 2020*	ResNet	Multi-view based dilated attention and residual network with sequential learning via convolutional LSTM	Spatial sequential information processing; Attention network to tackle class imbalance	Mean Dice = 0.8258	Private [LGE MRI, *n* = 190 (97 pre- and 93 post-ablation)]
	Chen et al., 2021	GAN	Adaptive attention cascade network for simultaneous estimation of unbalanced targets + joint discriminative network for adversarial regularization	Inter-cascade adversarial learning paradigm to tackle class imbalance and regularize the output	Mean Dice = 0.946	Private [LGE MRI, *n* = 192 (97 pre- and 95 post-ablation)]
(B) LV	Moccia et al., 2018	E-Net	E-Net	Relatively low accuracy; Unable to tackle class imbalance well	Dice = 0.55	Private (LV LGE MRI, *n* = 30)
	Moccia et al., 2019	FCNN	FCNN	Relatively low accuracy; Unable to tackle class imbalance well	Median Dice = 0.5400	Private (LV LGE MRI, *n* = 30)
	Zabihollahy et al., 2019a	CNN	Volume patch based 3D CNN	utilize small volume patches for accurate local view inspection	Mean Dice = 0.9363	Private (LV LGE MRI, *n* = 10)
	Fahmy et al., 2020	U-Net	U-Net based 3D CNN	Sub-volume design utilizes small volume patches for accurate local view inspection	Mean Dice = 0.54	Private (LV LGE MRI, multi-vendor *n* = 1073)

*As (Yang et al., 2020) and (Chen et al., 2018) reported very similar methodologies, we reported (Yang et al., 2020) only in this table.

#### 4.4.2 LA End-To-End Scar Segmentation Models

For LA (Table 6A), due to the thin LA wall, it is particularly difficult to achieve an end-to-end segmentation of scar directly from LGE CMR. A multi-view two task (MVTT) deep learning based method with dilated attention network was proposed to complete the two tasks simultaneously (Chen et al., 2018; Yang et al., 2020). This study also benchmarked a range of popular deep learning networks such as U-Net and V-Net on each of the two tasks. It compared the performance of its network with conventional methods such as 2-SD and k-means to demonstrate the superiority of its network in completing both of the two tasks accurately on both pre-ablation and post-ablation datasets (Yang et al., 2020). This study also suggested that 2-SD, k-means and fuzzy c-means methods clearly over-estimated the enhanced LA scar region (Yang et al., 2020).

Later, with a joint GAN discriminator, Chen et al. were able to further improve the segmentation accuracy by dealing with the significantly unbalanced two LA targets (LA wall and scar) (Chen et al., 2021; Table 7). In their method, cascaded learning, a widely applied technique in learning labels with unbalanced classes in natural image segmentation (Dai et al., 2016; Murthy et al., 2016; Li et al., 2017; Lin et al., 2017; Ouyang et al., 2017; Cai and Vasconcelos, 2018; Chen K. et al., 2019), demonstrated superiority in learning.

**TABLE 7:** Result of a private benchmarking (Chen et al., 2021) of different algorithms on the LASC’18 dataset, reported in their mean ± SD.

	LA and PVs		LA scar	
	Dice Scores	ASD (mm)	Dice Scores	ASD (mm)
2D U-Net	0.898 ± 0.034	3.38 ± 4.53	0.526 ± 0.118	1.83 ± 0.891
3D U-Net	0.895 ± 0.032	3.81 ± 3.89	0.508 ± 0.106	1.90 ± 0.837
MVTT (Yang et al., 2020)	0.902 ± 0.037	2.25 ± 1.39	0.613 ± 0.131	1.39 ± 1.03
JAS-GAN (Chen et al., 2021)	0.913 ± 0.027	2.24 ± 2.73	0.621 ± 0.110	1.24 ± 1.04

*ASD, average surface distance.*

#### 4.4.3 LV End-To-End Scar Segmentation Models

As LV has less variant morphology and greater size, there have been more successful methods demonstrating their efficacies andefficiencies in LV scar segmentation (Table 6B). E-Net (Moccia et al., 2018) and FCNN (Moccia et al., 2019) were the first few networks that demonstrated the ability to segment scar directly from LGE CMR. Although with relatively low Dice scores, they demonstrated that with an accurately segmented myocardium label it could perform better.

Recently, many deep learning methods have been proposed and demonstrated significantly higher efficacy compared to traditional threshold based methods. Zabihollahy et al. developed a CNN based network to classify each pixel by considering small volume patches around that pixel to greatly improve the mean segmentation accuracy in terms of its mean Dice score to 93.63, compared to the mean Dice scores of K-nearest neighbor (KNN) (77.85), FWHM (61.77), and 2SD (48.33) in their private benchmarking (Zabihollahy et al., 2019a).

In addition, Fahmy et al. (2020) proved that a 3D CNN deep learning based approach could be applied for LV scar segmentation for patients with hypertrophic cardiomyopathy (HCM) via a multicenter multivendor study.

Inspired by the two-stage approach, a multi-view cascaded U-Net driving for even higher efficacy in segmentation was developed to cascade the two tasks sequentially while considering sagittal, axial and coronal views (Moccia et al., 2019).

### 4.5 Segment LGE CMR Jointly With Other Modalities

As explained in Section 3.1.2, traditionally, clinicians check both bSSFP cine and LGE MRI modalities to ensure accurate segmentation of the myocardium and then the scar. Therefore, many methods suggested the use of both bSSFP cine and LGE modalities in delineating anatomical structures and scar to mimic that. For LA, it is also known that MRA gives a clear boundary in PV to help with LA wall segmentation. We can see many methods taking MRA as an extra modality into their models to enhance their segmentation accuracy. However, many studies chose bSSFP over MRA, as bSSFP can be acquired in the same phase as LGE CMR by cardiac gating. Although MRA provides better resolution, MRA is not cardiac gated and can be difficult and error-prone in co-registration with LGE CMR, causing misalignments in registered images. Additionally, as explained in Section 2.2, integration with other modality (e.g., T2) may enable more findings from the CMR (e.g., oedema) in addition to scars.

There are few challenges benchmarking a range of algorithms for the cross-modality fusion based segmentation of anatomy, scar and oedema. MS-CMR challenge (MS-CMR Challenge, 2019; Pop et al., 2020) presented a range of algorithms taking multiple modalities in to further improve the segmentation accuracy of LV myocardium, LV blood cavity and RV. MyoPS challenge (MyoPS Challenge, 2020; Zhuang and Li, 2020) presented algorithms to delineate LV myocardium with scarring and oedema.

Common methods to segment anatomy and scar from multiple modalities include:

(1) Cross-modality style and feature propagation (typically from bSSFP to LGE-MRI) [e.g., multi-atlas label fusion (MAS) (Zhu et al., 2017)].
(2) Combination of multiple paired sequences and modalities for segmentation by either cross-modality image style transfer [e.g., Cycle-GAN (Zhu et al., 2017) and UNIT style transfer (Huang et al., 2018; Chen J. et al., 2020)] or multi-input models [e.g., Multi-variable mixture model (MvMM) (Zhuang, 2019)].
(3) A two-stage approach to firstly co-registering anatomical segmentation from one modality to another (typically from bSSFP segmentation to LGE-MRI) and then segment scars based on the co-registered anatomy segmentation (Leong et al., 2019).

However, respiratory and/or cardiac motion complications between acquisitions of different modalities can still cause errors in registration and possible misalignments.

## 5. Scar Segmentation With Non-Contrast-Agent (Non-CA) Enhanced Imaging Modality Only

Although LGE CMR has been very successful in being the gold standard reference technique for AF and MI, including LGE in an MRI scanning significantly extends the scanning Time. Moreover, there have been increasingly growing concerns regarding the safety of the Gadolinium based contrast agent used, particularly for the patient with renal impairments (Ledneva et al., 2009). There has been a rising attention in exploring methods to segment scars without injecting contrast agents to the patients on non-CA modalities. Non-CA modality based cardiac scar segmentation methods have been widely demonstrated for LV scar delineations but has not been realized for LA scar delineations.

Dastidar et al. (2019) and Liu et al. (2018) demonstrated the potential of pre-contrast scar segmentation by comparing the inter-modality manual observations of myocardial infarction regions on LGE CMR and native-T1 mapping without the Gadolinium contrast agents.

### 5.1 Relaxation Time Based Scar Segmentation in T2

T1 and T2 (Messroghli et al., 2017) are modalities that are not enhanced by any contrast materials, where relaxation times in MI is longer compared to the healthy myocardium and could be referenced for MI region segmentation reproducibly (Abdel-Aty et al., 2004; Kali et al., 2014; Smulders et al., 2015). However, the relaxation time is field strength specific (Raman et al., 2013; Haaf et al., 2017) and requires the acquisition of images for additional breath holds, which significantly extends the CMR acquisition time.

### 5.2 MRI Feature Tracking

Magnetic resonance imaging feature tracking is also an approach to differentiate MI induced cardiac wall abnormalities from normal myocardium, which can be acquired as part of a standard CMR scanning examination (Muser et al., 2017; Ogawa et al., 2017). However, this technique can only detect and locate the position of MI without quantifying it.

### 5.3 Scar Segmentation in CINE MRI

To further improve scar segmentation on non-contrast enhanced CMR, trained by co-registered LGE and cine MRI modalities, SVM based texture analysis in pre-contrast cine MRI only can discriminate between nonviable, viable and remote segments (Larroza et al., 2018). Non-contrasted enhanced CMR scar segmentation has also been demonstrated via neighborhood approximation forests (Bleton et al., 2016), Simple Linear Iterative Clustering (SLIC) (Achanta et al., 2012) based supervoxels (Popescu et al., 2017).

#### 5.3.1 Deep Learning Based Scar Segmentation in CINE MRI

With the development of deep learning, a method based on a combination of Long short-term memory (LSTM), recurrent neural network (RNN) and fully convoluted neural network (FCNN) (Xu et al., 2017) and a GAN based method (Xu et al., 2018) have been demonstrated accuracy in detecting, locating and quantifying LV scarring regions from non-contrast enhanced CMR images. Zhang et al. proposed a deep learning based framework to greatly improve the efficacy of the segmentation of LV scar on cine MRI (with its stages consisting of (1) ROI localization, (2) RNN based motion pattern extraction, and (3) pixel classification by FCNN) and assess their network extensively under a clinical setting (Zhang et al., 2019). Xu et al. (2020) on top of the deep learning based workflow, proposed a progressive sequential causal generative adversarial network (GAN) to simultaneously synthesize LGE-equivalent images and multi-class tissue segmentation (including LV blood cavity, LV myocardium and scar region) from cine CMR images. A detailed summary and results of a private benchmarking of all these algorithms can be found in Table 8.

**TABLE 8:** Summary of representative machine learning/deep learning based scar segmentation in cine MRI for segmentation of cardiac scar regions on cine bSSFP MRI.

Reference	Method description	Pros/Cons	Private Benchmarking Accuracy (%) (Xu et al., 2020) (scar)	Dataset
Xu et al., 2020	(1) priori coarse tissue mask generation GAN, (2) condition LGE-equivalent image synthesis GAN, (3) fine segmentation GAN	Segment more than just LV scar – LV blood pool, myocardium and scar regions; Further improve temporal-spatial learning by a two-stream structure that includes a spatial perceptual pathway, a temporal perceptual pathway, and a multi-attention weighing unit.	97.13	Private [SAX cine bSSFP MRI, Xu et al. (2020), *n* = 280]
Zhang et al., 2019	(1) LV localization – ROI detection by CNN (2) Motion feature extraction (2.1) global motion feature – dense motion flow estimation (2.2) local motion feature – LSTM-RNN (3) infarction discrimination – FCNN	Combine both LSTM-RNN based local motion analysis and dense motion flow estimation based global motion analysis	95.03	
Xu et al., 2018	GAN (A) Generator: (A1) LV morphology and kinematic abnormalities – spatio-temporal feature extraction network through 3D successive convolution (A2) complementarity between segmentation and quantification - joint feature learning network for multitask learning; (B) Discriminator: (B1) intrinsic pattern between tasks – uses task relatedness network for adversarial learning	Introduce adversarial learning and task relatedness to reduce divergence	96.77	
Xu et al., 2017	(1) Heart localization – FAST R-CNN (Girshick, 2015) (2) Motion statistical feature – LSTM-RNN (3) discriminative layer – FCNN	Combine both ROI based local motion analysis and deep optical flow based global motion analysis	94.93	
Popescu et al., 2017	Simple Linear Iterative Clustering (SLIC) based supervoxels (Achanta et al., 2012)	Only radial strain analyzed, excluding longitudinal and circumferential strains; K-means clustering used requires an empirical definition of the number of clusters	86.47	
Bleton et al., 2016	Neighborhood approximation forests	Consider myocardial thickness and its temporal variations	84.39	

## 6. Evaluation Metrics

A range of evaluation metrics can be employed for assessing the results of the segmentation of the anatomy. These include Dice score, sensitivity, specificity, Hausdorff distance (HD) and surface-to-surface distance (STSD).

### (1) Dice Score

The Dice Score coefficient, DICE, is one of the most widely used evaluation metrics in segmentation accuracy evaluations. It is particularly sensitive to the difference between the ground truth label and the result label.

Given a 3D prediction label tensor, *A*, and 3D ground truth label tensor, *B*, the Dice score can be defined as:

(1) $$\text{DICE}\left(A,B\right)=\frac{2\left|\text{A}\cap \text{B}\right|}{\left|\text{A}\right|+\left|\text{B}\right|}$$

### (2) Sensitivity

Sensitivity score, also known as *True Positive Rate*, can be adapted to reflect the success of the algorithm for segmenting the foreground (cardiac anatomy) as:

(2) $$\text{Sensitivity}=\frac{\text{TP}}{\text{TP}+\text{FN}}$$

where *TP* stands for true positive and *FN* stands for false negative.

### (3) Specificity

Sensitivity score, also known as *True Negative Rate*, reflects the success of the algorithm for segmenting the background as:

(3) $$\text{Specificity}=\frac{\text{TN}}{\text{TN}+\text{FP}}$$

where *TN* stands for true negative and *FP* stands for false positive.

### (4) Hausdorff Distance

Hausdorff distance, HD, is an important parameter in evaluating the geometrical characteristics which measures the maximum local distance between the surfaces of the predicted LA volume label tensor, *A*, and the ground truth label tensor, *B*, given by:

(4) $$\text{HD}\left(A,B\right)=\max_{b\in B}\left\{\min_{a\in A}\left\{\sqrt{a^{2}-b^{2}}\right\}\right\}$$

where *a* and *b* are all pixels locations within *A* and *B*.

In practice, the HD is not generally recommended to use it directly since it has a great sensitivity to outliers, and as noises and outliers are quite common in medical image segmentation (Gerig et al., 2001; Zhang and Lu, 2004). However, Huttenlocher et al. (1993) proposed a way to handle outliers by defining the HD as the *q*^*th*^ quantile of distance instead of the maximum to exclude the outliers.

### (5) Surface-to-Surface Distance

Surface-to-surface distance, STSD, measures the average distance error between the surfaces of the predicted LA volume and the ground truth.

(5) STSD(A,B)=1nA+nB(∑p=1nAp2-B2+∑p′=1nBp′2-A2)

where *n*_*A*_ and *n_B_* are the numbers of pixels in A and B, respectively. Variables *p* and *p*′ describe all point between *A* and *B*.

The maximum error distance acceptable in the LA wall segmentation should be 1–2 mm under the clinical setting considering the thin LA wall (Xiong et al., 2021).

### (6) Error of the Anterior-Posterior Diameter of the Anatomical Structure

The anterior-posterior diameters of LA and LV are widely used as an essential clinical measure in clinical diagnosis and treatments.

The diameters can be estimated by finding the maximum Euclidean distance along the anterior-posterior axis of each CMR scan (Xiong et al., 2021).

### (7) Error of Volume of the Anatomical Structure

The anatomical volumes of LA and LV are widely used as an essential clinical measure in clinical diagnosis and treatments.

The volume of the structure can be found as the sum of positively labeled voxels. Given the volume of the predicted anatomical structure, *V_A_*, and the volume of the ground truth, *V_A_*, the total volume error can be defined as:

(6) $$\delta V=\left|V_{A}-V_{B}\right|$$

### (8) Scar Volume Percentage

In addition to the ones mentioned above, scar segmentation also employs a scar volume based metric in assessing the segmentation result, which is much more widely used as the quantification of scar is important for clinical use. They calculate the volumetric percentages of the scarring regions and compare them across the predicted and the ground truth labels.

The scar percentage is defined as the percentage of the volume of the scarring region, *V*_*scar*_, relative to the volume of the relevant anatomical wall, *V*_*wall*_ (e.g., LA wall) (Tao et al., 2010).

(7) $$\left[\%\right]scar=\frac{V_{scar}}{V_{wall}}\times 100\%$$

## 7. Discussion

### 7.1 Dataset Acquisition

#### 7.1.1 Inter-Observer Variability in the Manual Annotation of Ground Truth Labels

For validation and benchmarking of different methods and training of deep learning based methods, accurate, consistent and reproducible acquisition of ground truth labels is essential.

Validation by employing labels from a single clinician may not be ideal as these labels may exhibit bias and intra-observer variances when the same clinician is asked to repeat their labeling. Thus, it is recommended that we take observations from multiple clinicians and fuse them together.

However, we can see significant inter-observer variances, particularly for LA anatomical segmentation in LGE-MRI where the boundaries of the LA walls are very blurred. Kurzendorfer et al. (2017c) attempted to compensate for inter-observer variances by additional smoothing but ended up with slight improvement in Dice Scores (+0.04).

It is recommended that the data source reports the inter- and intra- observer variances by employing evaluation metrics such as the Dice Score coefficient. The currently widely used method of label fusing is obtaining a 70% consensus label among multiple annotations, which can be low in their consistency levels. The level of each observer’s expertise (novice, medical student, trainee, junior clinician or senior clinician) must also be clearly noted, particularly when multiple observers are involved. It may be also recommended that the observers should all be experienced senior clinicians to maintain the high accuracy and low variance in the manual annotation.

#### 7.1.2 Dataset Sources

Many methods use single-vendor single-center datasets to validate their methods, which may not demonstrate the ability to generalize the accurate segmentation methodology to centers with CMR machines of different settings and compositions.

There have been some trials assessing the performance of models based on multi-vendor and multi-center data (Engblom et al., 2016; Fahmy et al., 2020). However, evaluation based on multi-vendor and multi-center data with a more significant patient population should be introduced for a more comprehensive unbiased validation, comparison of performances of different methods and assessment for their scalability.

#### 7.1.3 Quantitative Result Reporting

We would like to alert readers that nearly all studies summarized in this study used their own distinct private datasets when reporting results. Biased conclusion may be derived when directly comparing these quantitative metrics across studies. The authors would like to ask all readers to refer to the experimental settings in acquisitions of the datasets stated in their original articles when comparing quantitative results across different studies, instead of only looking at these numbers stated. We would also look forward to a public benchmarking of all these methods as a fairer review of their performances.

Also, the authors would like to ask readers to be cautious when directly comparing Dice Scores reported for the segmentation of the LV infarction than the LA necrosis’. As the LA is much smaller than the LV, an equivalent volume of discrepancy may trigger a more significant reduction in the LA necrosis’ Dice Score ratios than the LV infarction’s. Additionally, the LA necrosis tends to be more challenging to be accurately segmented than the LV infarction explained above.

In addition, the image quality, contrast, class imbalance and other factors of the image data can directly impact the result generated from it and thus the accuracy reported. In particular, the authors would advocate future literature to report (1) scar to blood pool contrast ratios (SC-BP) (Karim et al., 2014) to show the scar contrast, (2) signal-to-noise ratio (SNR) to show the noise variation along with evaluation metrics in results, so the readers can have a better understanding of the experimental settings before interpreting all the metrics reported quantitatively. These two additional metrics are essential, particularly when it comes to LA scar segmentation, where the scar segmentation is more difficult and where higher SC-BP can give higher Dice Scores in the results generated (Karim et al., 2014).

### 7.2 Conventional Methods

#### 7.2.1 Advantages – Computational Load and Explainability

Obviously, as conventional methods are less demanding on the composition of the computing device, they can be deployed for wider clinical uses more easily. This is an advantage when it comes to the scalability and generalizability of the product, where a standard computer is enough for its deployment.

Conventional methods are also more explainable than deep learning. The explainability also guarantees easier acceptance from the clinicians, as the product may appear more trustworthy and more reliable.

#### 7.2.2 How Reliable Are the Conventional Methods?

##### 7.2.2.1 Fixed threshold conventional methods

Fixed threshold methods may not fit some LGE CMR images, as they are unlikely to handle variations well (Oakes et al., 2009).

Scars are highly variable in their morphology and their brightness distribution on LGE CMR. Some severe LV cardiac scar may appear bright in its surroundings and very dark in its center, as the center of the scar is so severely infarcted that very little GBCA carrying perfusion arrives there. N-SD and FWHM, which require the pixel intensity to be more than a certain threshold for that pixel to be recognized as a scar, may not label these dark centers as the scar. Additionally, due to the partial volume effect, fibrotic regions containing both intermingling bundles of fibrotic and viable myocytes will be darker than the complete necrosis region. The low intensity exhibited from such fibrotic regions may be below the fixed threshold set and make these fibrotic regions be falsely recognized as healthy myocardium.

Varied external factors including resolution, contrast, signal-to-noise ratio (SNR), inversion time and surface coil intensity variation can also adversely affect the accuracy of the scar segmentation. LGE CMR modality often suffers from poor image quality, which may be due to residual respiratory motion, variability in the heart rate and gadolinium wash-out during the currently long acquisition time (Yang et al., 2017). Considering the thin transmural thickness of the atrial wall [mean = 2.2–2.5 mm (Saìnchez-Quintana et al., 2005)] (Figure 2), the spatial resolution of LGE CMR images is relatively limited, particularly for the left atrium (To et al., 2011). The variable anatomical morphological shapes of pulmonary veins (PV) also impose an additional challenge to the LGE CMR segmentations. In addition, some uninterested cardiac substructures may be highlighted in LGE CMR images as well in addition to the scarring and fibrosis regions. These may be due to the navigator beam artifact (which is often seen near the right PV), Gadolinium uptake by the aortic wall and valves and confounded enhancement in the spine, esophagus, etc. (Karim et al., 2013; Yang et al., 2017).

##### 7.2.2.2 Conventional adaptive methods

Although adaptive conventional methods may mitigate adverse impacts from variable scar shapes and varied external factors, adaptive conventional methods can also be affected by sizes, variances and artifacts in testing image data as they utilize prior information learned. Kurzendorfer et al. (2017c) showed that a particular scar distribution over the myocardium could adversely affect their methods in segmenting endocardial contours. Such vulnerability may be more problematic when it comes to LA anatomical structures, as PV is a very morphological variable and LA walls are much smaller and thinner.

### 7.3 Deep Learning Based Methods

#### 7.3.1 How We Could Make the Deep Learning Perform Even Better?

For detailed designs of the deep learning networks, LASC’18 benchmarked (Xiong et al., 2021) a range of U-Net variants in LA wall segmentation from LGE CMR. This challenge, along with other literature for cardiac scar segmentation, demonstrated the following.

(1) Image Sources

(a) Higher image qualities (as in signal-to-noise ratio) would result in a higher Dice Score, although not statistically significantly linearly related.
(b) In addition, models with contrast normalization as a pre-processing technique performed significantly better than the ones without using normalization.

(2) Model Backbone

(a) CNN based methods delivered better results compared to the atlas based methods.
(b) U-Net based methods outperformed other networks using VGGNet, ResNet, etc.
(c) There was no statistical difference between the segmentation performances of the models based on 2D CNNs and models based on 3D CNNs. However, further research showed that 3D CNNs greatly outperformed 2D CNNs with the same model architecture in terms of the Dice Scores of their segmentation results (Borra et al., 2020a).

(3) Segment on ROI or the Whole Image?

(a) Centring LA on ROI as an input to the second sequential model would make the model perform significantly better compared to the model with non-centered ROIs.
(b) Class imbalance induced by significantly big or small ROI size could lead to an adverse effect on the segmentation results in terms of Dice scores.
(c) Double sequential CNNs (Li et al., 2019; Xia et al., 2019; Yang et al., 2019; Xiong et al., 2021) (one detecting the region of interest first and then the second model performing regional segmentation within the region of interests (ROI) detected) achieved much better results compared to the methods with only one single CNN.
(d) Double sequential 3D CNN outperformed single 2D CNN and single 3D CNN models regarding its Dice scores, surface distance, LA diameter error and LA volume error.

(4) Model Architecture

(a) Models with residual connections performed significantly better compared to the ones without residual connections.
(b) The use of dropout blocks did not perform significantly better than the one without using dropout.
(c) Rectified Linear Unit (ReLU) trained models did not perform significantly better than the Parametric Rectified Linear Unit (PReLU) trained models.

(5) Loss Functions

(a) Dice loss trained models performed significantly better than the cross-entropy trained models.

#### 7.3.2 Problems With Deep Learning in Segmentation

##### 7.3.2.1 Computational load

Although we are able to observe much better results generated from deep learning based methods, we can also observe a rise in computational demand from deep learning networks. For deep learning based methods, high-end computer graphics processing units (GPUs) become a necessity when deploying these models, whereas standard computers with CPUs only are sufficient for most of the conventional methods to run. Under a clinical setting, hiring a GPU is not always possible, as it is not part of a standard clinical computing workstation. The requirement of a high-end computer with GPU in deploying a deep learning based method may significantly limit the ability of these methods to scale.

However, if a standard computer was only used to infer a deep learning model, its runtime may be a bit long but still falls within the maximum time limit that clinicians can accept (usually a few minutes per slice for models that are not extra complex). Therefore, we can see these models can be deployed and scaled only if they are sophisticatedly trained, as training on the clinician’s side, where unlikely they have a GPU, is not usually possible. As the inference time may vary significantly across different models over CPUs and depend on their architectures and complexities, reporting of inference time per slice on a standard computer without a GPU should also be mandatory in addition to the inference time over a GPU.

##### 7.3.2.2 Scarcity of annotated data

Training datasets with abundant paired labels are essential to the success of deep learning model training. However, there has been a scarcity of labels due to the tedious process of manually annotating the ground truths in medical imaging. In order to mitigate such scarcity in ground truth labels, several methods can be adopted, including the following.

(1) Data augmentation,
(2) Transfer learning with fine-tuning (Bai et al., 2018; Chen S. et al., 2019; Khened et al., 2019),
(3) Weak and semi-supervised learning (Bai et al., 2017, 2018; Can et al., 2018; Chartsias et al., 2018, 2019; Kervadec et al., 2019),
(4) Self-supervised learning (Bai et al., 2019) and,
(5) Unsupervised learning (Joyce et al., 2018).

In addition, to mitigate the challenging training process brought by the great data size required to train a scalable network, active learning (Mahapatra et al., 2018) has been introduced to reduce manual annotation workloads as well as the computational loads.

##### 7.3.2.3 Explainability in deep learning

Although there has been a wide range of evidence demonstrating the efficacy of deep learning in medical image analysis, the deep learning networks behave more like a ‘black box,’ where its interpretability is poor. It has been shown that these deep learning networks can be attacked by adversarial noises or even just rotation in medical images (Finlayson et al., 2019), questioning the reliability and scalability of these deep learning models in assisting diagnosis. For alerting users of these possible failures, segmentation quality scores (Robinson et al., 2019) and confidence maps [e.g., uncertainty maps (Sander et al., 2019) and attention maps (Heo et al., 2018)] should be provided to highlight uncertainties in the model prediction.

### 7.4 Non-CA Modality Segmentation: Bye-Bye to Gadolinium?

Although many methods can accurately segment scars on non-CA cine MRI, the impact from different numbers of cardiac phases on cine MRI has not been assessed.

In addition, the binary class of either normal or scar may be too simplistic. Quantification of the so-called “gray-zone,” which has been proposed for the clinical implication of ventricular arrhythmia (Jablonowski et al., 2017), immediately surrounding the ventricular scar may be useful clinically.

Also, gadolinium based contrast agent is not only applied for scar imaging but also for assessing myocardial perfusion, which is usually assessed together in LGE CMR, for which additional classification and differentiation of ischemic and remote regions of myocardium would be useful (Leiner, 2019). To achieve that, Liu et al. (2016) demonstrated non-Gadolinium contrast adenosine stress and rest T1 Mapping for identification and classification of normal, infarcted, ischemic and remote regions in LV myocardium.

We are glad to see a range of algorithms demonstrated for LV scar segmentation in non-contrast enhanced CMR. However, this has not been realized for CMR images of LA, which is more difficult as the LA scarring regions in CMR suffers from greater variances in morphology and relatively lower resolution of CMR. Moreover, LA scars can appear in discrete regions (Figure 2), which imposes further challenges to the LA scar segmentation from non-CA modalities.

## Conclusion

This study summarizes the recent developments in cardiac scar segmentation, covering a wide range of conventional and deep learning techniques. In particular, we presented and discussed the usefulness of non-LGE modalities in cardiac anatomy and scar segmentation. We then further discussed the recent progress in segmenting the cardiac scarring region from non-contrast-enhanced images. We hope this review can provide a comprehensive understanding of the segmentation methodologies for cardiac scar and fibrosis and increase the awareness of common challenges in these fields that can call for future research and contributions.

## Author Contributions

YW and GY contributed to study design and writing – original draft preparation. ZT, BL, YW, and GY contributed to data collection. YW, ZT, BL, and GY contributed to data visualization. YW, ZT, BL, DF, and GY contributed to writing – review and editing. DF and GY contributed to supervision and funding acquisition. All authors have read and agreed to the published version of the manuscript.

## Conflict of Interest

The authors declare that the research was conducted in the absence of any commercial or financial relationships that could be construed as a potential conflict of interest.

## Publisher’s Note

All claims expressed in this article are solely those of the authors and do not necessarily represent those of their affiliated organizations, or those of the publisher, the editors and the reviewers. Any product that may be evaluated in this article, or claim that may be made by its manufacturer, is not guaranteed or endorsed by the publisher.
